# Extended Clavien-Dindo classification of surgical complications: Japan Clinical Oncology Group postoperative complications criteria

**DOI:** 10.1007/s00595-015-1236-x

**Published:** 2015-08-20

**Authors:** Hiroshi Katayama, Yukinori Kurokawa, Kenichi Nakamura, Hiroyuki Ito, Yukihide Kanemitsu, Norikazu Masuda, Yasuhiro Tsubosa, Toyomi Satoh, Akira Yokomizo, Haruhiko Fukuda, Mitsuru Sasako

**Affiliations:** Japan Clinical Oncology Group Data Center/Operations Office, Center for Research Administration and Support, National Cancer Center, Tokyo, Japan; Department of Gastroenterological Surgery, Osaka University Graduate School of Medicine, Suita, Japan; Department of Thoracic Surgery, Kanagawa Cancer Center, Yokohama, Japan; Colorectal Surgery Division, National Cancer Center Hospital, Tokyo, Japan; Department of Surgery, Breast Oncology, National Hospital Organization, Osaka National Hospital, Osaka, Japan; Division of Esophageal Surgery, Shizuoka Cancer Center, Shizuoka, Japan; Department of Obstetrics and Gynecology, Faculty of Medicine, University of Tsukuba, Tsukuba, Japan; Department of Urology, Graduate School of Medical Sciences, Kyushu University, Fukuoka, Japan; Department of Surgery, Hyogo College of Medicine, Nishinomiya, Japan

**Keywords:** JCOG postoperative complications criteria (JCOG PC criteria), Clavien-Dindo classification, Postoperative complications

## Abstract

**Purpose:**

Prior to publication of the Clavien-Dindo classification in 2004, there were no grading definitions for surgical complications in either clinical practice or surgical trials. This report establishes supplementary criteria for this classification to standardize the evaluation of postoperative complications in clinical trials.

**Methods:**

The Japan Clinical Oncology Group (JCOG) commissioned a committee. Members from nine surgical study groups (gastric, esophageal, colorectal, lung, breast, gynecologic, urologic, bone and soft tissue, and brain) specified postoperative complications experienced commonly in their fields and defined more detailed grading criteria for each complication in accordance with the general grading rules of the Clavien-Dindo classification.

**Results:**

We listed 72 surgical complications experienced commonly in surgical trials, focusing on 17 gastroenterologic complications, 13 infectious complications, six thoracic complications, and several other complications. The grading criteria were defined simply and were optimized for surgical complications.

**Conclusions:**

The JCOG postoperative complications criteria (JCOG PC criteria) aim to standardize the terms used to define adverse events (AEs) and provide detailed grading guidelines based on the Clavien-Dindo classification. We believe that the JCOG PC criteria will allow for more precise comparisons of the frequency of postoperative complications among trials across many different surgical fields.

## Introduction

The evaluation of postoperative complications in surgical trials is as important as the assessment of toxicities in chemotherapy trials. Prior to the proposal of a therapy-oriented classification scheme, by Clavien PA et al. in 1992 [1], there were no accepted definitions for the grading of surgical complications in clinical practice. This framework proposed by Clavien et al. was not used widely, because there was no system for the grading of severity of surgical complications [2] and no uniform definition of these events. For instance, some surgeons included a body temperature greater than 38 °C on two consecutive days as being “high”, whereas others included intraoperative complications, postoperative complications (within 30 days), and late events such as dumping syndrome. Few randomized controlled trials (RCTs) [3] have used this classification system, with individual parochial definitions of surgical complications being used in most surgical RCTs [4–6].

In cancer clinical trials, adverse events (AEs) are evaluated in accordance with the Common Terminology Criteria for Adverse Events (CTCAE), which is far from exhaustive in terms of surgical complications; thus, some surgeons are not comfortable using grading definitions. The Clavien-Dindo classification, published in 2004 [7] defined a simple classification of postoperative complications, which has been adopted widely in clinical practice. Although this classification categorizes postoperative complications broadly into four major groups, it is often desirable to more clearly define the common AEs to avoid the use of different or less precise terms for the same AEs occurring in different clinical trials. More detailed grading criteria for common AEs would also be helpful for surgeons. Therefore, our aim was to establish supplementary criteria for the Clavien-Dindo classification to standardize the evaluation of postoperative complications.

## Methods

The Japan Clinical Oncology Group (JCOG) commissioned a committee to establish more precise criteria for the grading of surgical complications. The committee comprised members from nine JCOG study groups (gastric, esophageal, colorectal, lung, breast, gynecologic, urologic, bone and soft tissue, and brain) who have extensive experience with surgical trials. These groups established the JCOG postoperative complications criteria (JCOG PC criteria). Members identified the postoperative complications experienced commonly in their fields and defined detailed grades for each complication in accordance with the general grading rules of the Clavien-Dindo classification. The JCOG PC criteria were reviewed and approved by the JCOG Executive Committee and published on the JCOG website in October, 2011 (in Japanese) [8].

## Results

The JCOG PC criteria included 72 surgical AEs experienced commonly in surgical trials, including 17 gastroenterological complications, 13 infectious complications, six thoracic complications, and several other complications (Table 1). If no applicable AE terms are found in the JCOG PC criteria, ‘other (specify)’ should be chosen. In such cases, the appropriate AE term should be used, and the overall grading should be performed in accordance with the general rules of the Clavien-Dindo classification. Because the grading definitions follow the general rules of the Clavien-Dindo classification, surgeons can use these original rules to grade AEs, and can also refer to the more detailed definitions in the JCOG PC criteria if necessary. Table 2 lists the differences between CTCAE, the Clavien-Dindo classification, and the JCOG PC criteria.

**Table 1:** List of surgical adverse event (AE) terms and gradings

	Principle of grading							
	I	II	IIIa	IIIb	IVa	IVb	V	Supplemental explanation of suffix “d”
AE term	Any deviation from the normal postoperative course without the need for pharmacological treatment or surgical, endoscopic, or radiological interventions. Allowed therapeutic regimens include drugs such as antiemetics, antipyretics, analgesics, diuretics, electrolytes, and physiotherapy. This grade also includes wound infections opened at the bedside	Requirement for pharmacological treatment with drugs other than those allowed for grade I complications Blood transfusions and total parenteral nutrition are also included	Requirement for surgical, endoscopic or radiological intervention not under general anesthesia	Requirement for surgical, endoscopic or radiological intervention under general anesthesia	Life-threatening complications (including CNS complications)* requiring IC/ICU management. Single organ dysfunction (including dialysis)	Life-threatening complications (including CNS complications)* requiring IC/ICU management. Multiple organ dysfunction	Death of the patient	If the patient suffers from a complication at the time of discharge, the suffix “d” (for “disability”) is added to the respective grade of complication. This label indicates the need for a follow-up to fully evaluate the complication
Stroke	Clinical observation only; intervention not indicated	Medical management indicated (e.g., anticoagulant therapy)	Radiological intervention without general anesthesia (e.g., intracerebrovascular treatment)	Intervention under general anesthesia indicated (e.g., drainage, surgical clipping, cerebrovascular bypass, carotid endarterectomy)	IC/ICU management indicated	IC/ICU management indicated; associated with respiratory failure	Death	Persistent hemiplegia
Recurrent laryngeal nerve palsy	Clinical observation or diagnostic evaluation only; intervention not indicated	Aspiration; medical management indicated (e.g., antibiotics)	Severe aspiration; food intake almost impossible; medical intervention under local anesthesia indicated (e.g., vocal cord injection, tracheal puncture)	Intervention under general anesthesia indicated (including tracheostomy under sedation)	Mechanical ventilation indicated	Sepsis or multiple organ failure	Death	Hoarseness, difficulty in speaking; communication through writing necessary; discharged with tracheostomy
Upper extremity paresthesia	Clinical observation only; intervention not indicated	Medical management indicated	Surgical intervention without general anesthesia indicated (e.g., nerve block)	–	–	–	–	Persistent brachial paresthesia
Paresthesia in resected part (Phantom pain)	Clinical observation only; intervention not indicated	Medical management indicated	Surgical intervention without general anesthesia indicated (e.g., nerve block)	–	–	–	–	Persistent phantom pain
Ischemic heart disease	Clinical observation or diagnostic evaluation only; intervention not indicated	Medical management indicated (e.g., anticoagulant therapy)	Cardiac catheterization indicated	Intervention under general anesthesia indicated (coronary artery bypass)	Heart failure associated with low cardiac output syndrome; IC/ICU management indicated	Heart failure associated with low cardiac output syndrome and renal failure; IC/ICU management indicated	Death	Persistent heart failure following myocardial infarction
Pericardial effusion	Clinical observation or diagnostic evaluation only; intervention not indicated (drainage only through existing drainage tube)	Medical management indicated	Image-guided drain placement/paracentesis including drain replacement indicated	Intervention under general anesthesia indicated (fenestration)	Cardiac tamponade; IC/ICU management indicated	Cardiac tamponade and renal failure; IC/ICU management indicated	Death	–
Bradyarrhythmia	Clinical observation or diagnostic evaluation only; intervention not indicated	Medical management indicated (e.g., atropine, β agonists)	Medical intervention under local anesthesia indicated (e.g., pacemaker implantation)	–	Heart failure associated with low cardiac output syndrome; IC/ICU management indicated	Heart failure associated with low cardiac output syndrome and renal failure; IC/ICU management indicated	Death	–
Supraventricular arrhythmia	Clinical observation or diagnostic evaluation only; intervention not indicated	Medical management indicated (e.g., antiarrhythmic drugs)	Medical intervention under local anesthesia indicated (e.g., catheter ablation, synchronized cardioversion)	–	Heart failure associated with low cardiac output syndrome; IC/ICU management indicated	Heart failure associated with low cardiac output syndrome and renal failure; IC/ICU management indicated	Death	–
Ventricular arrhythmia	Clinical observation or diagnostic evaluation only; intervention not indicated	Medical management indicated (e.g., antiarrhythmic drugs)	Medical intervention under local anesthesia indicated (e.g., catheter ablation, external defibrillator, pacemaker implantation)	–	Heart failure associated with low cardiac output syndrome; IC/ICU management indicated	Heart failure associated with low cardiac output syndrome and renal failure; IC/ICU management indicated	Death	–
Atelectasis/sputum excretion difficulty	Clinical observation or diagnostic evaluation only; intervention not indicated, except for nebulizers, expectorants, or lung physiotherapy (e.g., postural drainage)	Medical management indicated (e.g., antibiotics)	Bronchoscopic aspiration or surgical intervention indicated (e.g., tracheal puncture) without general anesthesia	Intervention under general anesthesia indicated (including tracheostomy under sedation)	Mechanical ventilation indicated	Sepsis or multiple organ failure	Death	Discharged with tracheostomy
Tracheal fistula, bronchial fistula	Clinical observation or diagnostic evaluation only; intervention not indicated	–	Procedure under local anesthesia indicated	Intervention under general anesthesia indicated	Mechanical ventilation indicated	Sepsis or multiple organ failure	Death	Discharged with tube drainage, open drainage
Pulmonary fistula	Clinical observation or diagnostic evaluation only; intervention not indicated (drainage only through existing drainage tube)	–	Procedure under local anesthesia indicated (e.g., chest tube drainage, pleurodesis) including drain replacement indicated.	Intervention under general anesthesia indicated (Closure for pleuroparenchymal defects, pleurodesis)	Mechanical ventilation indicated	Sepsis or multiple organ failure	Death	Discharged with tube drainage, open drainage
Chylothorax	Observation of chylous drainage fluid or thoracentesis fluid only (drainage only through existing drainage tube)	Fat-restricted diet, intravenous nutrition indicated	Image-guided drain placement/paracentesis including drain replacement indicated	Intervention under general anesthesia indicated (e.g., thoracic duct ligation)	–	–	Death	Persistent respiratory distress, malnutrition
Pleural effusion	Clinical observation or diagnostic evaluation only; intervention not indicated (drainage only through existing drainage tube)	Medical management indicated (e.g., diuretics)	Image-guided drain placement/thoracentesis including drain replacement indicated	Intervention under general anesthesia indicated	Mechanical ventilation indicated	Multiple organ failure	Death	Persistent respiratory distress
Lung torsion	–	–	–	Intervention under general anesthesia indicated (e.g., detorsion, lobectomy)	Mechanical ventilation indicated	Sepsis or multiple organ failure	Death	–
Ascites	Clinical observation or diagnostic evaluation only; intervention not indicated (drainage only through existing drainage tube)	Medical management indicated (e.g., diuretics)	Image-guided drain placement/paracentesis including drain replacement indicated	Intervention under general anesthesia indicated	–	–	Death	Persistent abdominal fullness
Diarrhea	Intestinal fluid excretion ≥2000 ml/day; intervention not indicated	Intestinal fluid excretion ≥2000 ml/day associated with dehydration or electrolyte abnormality; intravenous fluids indicated	–	–	At least one organ failure (e.g., pulmonary disorders requiring mechanical ventilation or nephropathy indicating dialysis)	Sepsis or multiple organ failure	Death	Significant amount of persistent intestinal fluid excretion
Dysphagia	Clinical observation only; intervention not indicated	Enteral/intravenous nutrition (Including TPN) indicated	Medical intervention under local anesthesia indicated (e.g., tracheal puncture, endoscopic gastrostomy)	Intervention under general anesthesia indicated	–	–	Death	Gastrostomy
Intestinal fistula	Clinical observation or diagnostic evaluation only; intervention not indicated (drainage only through existing drainage tube)	Medical management indicated (e.g., antibiotics)	Image-guided drain placement/paracentesis including drain replacement indicated	Intervention under general anesthesia indicated (colostomy)	At least one organ failure (e.g., pulmonary disorders requiring mechanical ventilation or renal disorders indicating dialysis)	Sepsis or multiple organ failure	Death	Persistent enterocutaneous fistula
Intestinal ischemia/necrosis	Clinical observation or diagnostic evaluation only; intervention not indicated	Medical management indicated (e.g., antibiotics)	Radiological intervention/endoscopic/surgical intervention without general anesthesia indicated	Intervention under general anesthesia indicated (e.g., intestinal resection)	At least one organ failure (e.g., pulmonary disorders indicating mechanical ventilation or renal disorders indicating dialysis)	Sepsis or multiple organ failure	Death	Home enteral/intravenous nutrition
Gastric tube necrosis	Observation of a small fistula with oral contrast study or drainage imaging (drainage only through existing drainage tube)	Medical management (e.g., antibiotics), enteral/intravenous nutrition indicated	Radiological intervention/endoscopic/elective surgical intervention without general anesthesia indicated, including drain replacement	Intervention under general anesthesia indicated	At least one organ failure (e.g., pulmonary disorders requiring mechanical ventilation or nephropathy indicating dialysis)	Sepsis or multiple organ failure	Death	
Reflux esophagitis	Clinical observation or diagnostic evaluation only; intervention not indicated	Medical management (e.g., PPI, pancreatic enzyme inactivators) or enteral/intravenous nutrition indicated	–	Intervention under general anesthesia indicated	–	–	Death	Persistent heartburn
Ileus (paralytic)	Clinical observation or diagnostic evaluation only; medical management not indicated except for laxatives and intravenous nutrition	Medical management beyond laxatives, NG tube placement, or intravenous nutrition management indicated	Nasoenteric tube placement	Treatment for ileus under general anesthesia (with or without intestinal resection)	Extensive intestinal necrosis, at least one organ failure (e.g., pulmonary disorders requiring mechanical ventilation or nephropathy indicating dialysis)	Sepsis or multiple organ failure	Death	Home intravenous nutrition
Pancreatic fistula	On or after postoperative day 3, drainage fluid amylase level ≥3 times the upper limit of institutional criteria, but intervention not indicated	Medical management indicated (e.g., antibiotics), enteral/intravenous nutrition indicated	Image-guided drain placement/paracentesis including drain replacement indicated	Intervention under general anesthesia indicated	At least one organ failure (e.g., pulmonary disorders requiring mechanical ventilation or nephropathy indicating dialysis)	Sepsis or multiple organ failure	Death	Residual pancreatic pseudocyst on CT, occasional fever, or abdominal pain
Intestinal obstruction	Clinical observation or diagnostic evaluation only; medical management not indicated except for laxatives and intravenous nutrition	Medical management beyond laxatives, NG tube placement, or intravenous nutrition management indicated	Nasoenteric tube placement	Treatment for ileus under general anesthesia (with or without intestinal resection)	Extensive intestinal necrosis, at least one organ failure (e.g., pulmonary disorders requiring mechanical ventilation or nephropathy indicating dialysis)	Sepsis or multiple organ failure	Death	Home intravenous nutrition
Delayed gastric emptying	Clinical observation or diagnostic evaluation only; intervention not indicated	Medical management (e.g., peristalsis stimulating drugs), NG tube placement, enteral/intravenous nutrition indicated	–	Intervention under general anesthesia indicated	–	–	Death	Persistent postprandial nausea
Dumping syndrome	Clinical observation only; intervention not indicated	Medical management indicated	–	Intervention under general anesthesia indicated	–	–	Death	Persistent dumping symptom
Biliary fistula	Clinical observation or diagnostic evaluation only; intervention not indicated (drainage only through existing drainage tube)	Medical management indicated (e.g., antibiotics)	Image-guided drain placement/paracentesis including drain replacement indicated	Intervention under general anesthesia indicated (drainage)	At least one organ failure (e.g., pulmonary disorders requiring mechanical ventilation or nephropathy indicating dialysis)	Sepsis or multiple organ failure	Death	Residual pseudocyst on CT; occasional fever or abdominal pain
Cholecystitis	Clinical observation or diagnostic evaluation only; medical management not indicated except for cholagogues	Medical management beyond cholagogues indicated	Medical intervention under local anesthesia indicated (e.g., Percutaneous transhepatic gallbladder drainage)	Intervention under general anesthesia indicated (cholecystectomy)	At least one organ failure (e.g., pulmonary disorders requiring mechanical ventilation or nephropathy indicating dialysis)	Sepsis or multiple organ failure	Death	Occasional fever or abdominal pain
Gastrointestinal anastomotic leak	Only small fistula observed on oral contrast study or drainage imaging (drainage only through existing drainage tube)	Medical management (e.g., antibiotics) or enteral/intravenous nutrition (Including TPN) indicated	Image-guided drain placement/paracentesis including wound opening or drain replacement indicated	Intervention under general anesthesia indicated (e.g., suture, reanastomosis, bypass, drainage, colostomy)	At least one organ failure (e.g., pulmonary disorders requiring mechanical ventilation or nephropathy indicating dialysis)	Sepsis or multiple organ failure	Death	Home enteral/intravenous nutrition
Ureteric injury	Clinical observation or diagnostic evaluation only; intervention not indicated	Medical management indicated (e.g., antibiotics)	Transurethral ureteral stent insertion or percutaneous nephrostomy	Intervention under general anesthesia indicated	Acute renal failure, hemodialysis	Sepsis or multiple organ failure	Death	Discharged with ureteral stent
Urethral injury	Foley catheter placement	Medical management indicated (e.g., antibiotics)	Intervention under local or lumbar anesthesia indicated (e.g., percutaneous cystostomy)	Intervention under general anesthesia indicated	At least one organ failure (e.g., pulmonary disorders requiring mechanical ventilation or nephropathy indicating dialysis)	Sepsis or multiple organ failure	Death	Discharged with Foley catheter placement
Postoperative hemorrhage	Controllable with compression only	Blood transfusion or medical management indicated	Surgical hemostasis under local anesthesia or endoscopic and radiological intervention hemostasis indicated	Intervention under general anesthesia indicated (hemostasis)	Single organ failure; stepdown ICU/ICU care indicated	Multiple organ failure; IC/ICU management indicated	Death	Persistent anemia
Seroma (Accumulation of serous fluid)	Bedside paracentesis only (drainage only through existing drainage tube)	–	Image-guided drain placement/paracentesis including drain replacement indicated	Intervention under general anesthesia indicated	At least one organ failure (e.g., pulmonary disorders requiring mechanical ventilation or nephropathy indicating dialysis)	Sepsis or multiple organ failure	Death	Exudate leakage from wound, occasional fever and infection, discharged with drainage tube
Uterine anastomotic leak	Clinical or vaginal observation only; intervention not indicated	Medical management indicated (e.g., antibiotics)	–	Intervention under general anesthesia indicated (resuturing)	At least one organ failure (e.g., pulmonary disorders requiring mechanical ventilation or nephropathy indicating dialysis)	Sepsis or multiple organ failure	Death	Persistent leakage from uterovaginal anastomosis due to suture failure (surgical union of two different anatomical structures)
Abdominal incisional hernia	Clinical observation only; intervention not indicated except for truss and NSAIDs	Medical management beyond truss and NSAIDs indicated	Medical intervention under local anesthesia indicated	Intervention under general anesthesia indicated (mesh, fascial resuturing)	Extensive intestinal necrosis, at least one organ failure (e.g., pulmonary disorders requiring mechanical ventilation or nephropathy indicating dialysis)	Sepsis or multiple organ failure	Death	Intestinal prolapse upon increased intra-abdominal pressure
Wound dehiscence	Clinical observation only; intervention not indicated except for wound irrigation	Medical management indicated (e.g., antibiotics)	Medical intervention under local anesthesia indicated (e.g., resuturing)	Intervention under general anesthesia indicated (e.g., resuturing)	Extensive intestinal necrosis, at least one organ failure (e.g., pulmonary disorders requiring mechanical ventilation or nephropathy indicating dialysis)	Sepsis or multiple organ failure	Death	Discharged with significant wound dehiscence
Gastrointestinal anastomotic stenosis	Clinical observation or diagnostic evaluation only; intervention not indicated	Enteral/intravenous nutrition (Including TPN) indicated	Balloon dilatation, stenting, magnetic compression anastomosis	Intervention under general anesthesia indicated (e.g., reanastomosis, bypass)	–	–	Death	Frequent outpatient endoscopic dilatation
Intraabdominal abscess	Clinical observation or diagnostic evaluation only; intervention not indicated	Medical management indicated (e.g., antibiotics)	Image-guided drain placement/paracentesis including drain replacement indicated	Intervention under general anesthesia indicated (drainage)	At least one organ failure (e.g., pulmonary disorders requiring mechanical ventilation or nephropathy indicating dialysis)	Sepsis or multiple organ failure	Death	Residual abscess on CT, occasional fever or abdominal pain
Pelvic abscess	Clinical observation or diagnostic evaluation only; intervention not indicated	Medical management indicated (e.g., antibiotics)	Image-guided drain placement/paracentesis including drain replacement indicated	Intervention under general anesthesia indicated (drainage)	At least one organ failure (e.g., pulmonary disorders requiring mechanical ventilation or nephropathy indicating dialysis)	Sepsis or multiple organ failure	Death	Residual abscess on CT, occasional fever or abdominal pain
Pneumonia	Clinical observation or diagnostic evaluation only; intervention not indicated except for nebulizers, expectorants, or lung physiotherapy (e.g., postural drainage)	Medical management indicated (e.g., antibiotics)	Bronchoscopic aspiration, tracheal puncture	Tracheostomy under general anesthesia/sedation or mechanical ventilation	Mechanical ventilation indicated	Sepsis or multiple organ failure	Death	Persistent respiratory distress, occasional fever
Mediastinitis	Clinical observation or diagnostic evaluation only; intervention not indicated	Medical management indicated (e.g., antibiotics)	Image-guided drain placement/paracentesis including drain replacement indicated	Intervention under general anesthesia indicated (drainage)	At least one organ failure (e.g., pulmonary disorders requiring mechanical ventilation or nephropathy indicating dialysis)	Sepsis or multiple organ failure	Death	Residual abscess on CT images, occasional fever or abdominal pain
Pyothorax	Clinical observation or diagnostic evaluation only; intervention not indicated	Medical management indicated (e.g., antibiotics)	Image-guided drain placement/paracentesis including drain replacement indicated	Intervention under general anesthesia indicated (drainage)	At least one organ failure (e.g., pulmonary disorders requiring mechanical ventilation or nephropathy indicating dialysis)	Sepsis or multiple organ failure	Death	Residual abscess on CT images or discharged with tube drainage, open drainage
Lower extremity lymphangitis (Lymph node infection)	Clinical observation or diagnostic evaluation only; intervention not indicated	Medical management indicated (e.g., antibiotics)	Medical intervention under local anesthesia indicated (lymphatic anastomosis)	Intervention under general anesthesia indicated (lymphatic anastomosis)	At least one organ failure (e.g., pulmonary disorders requiring mechanical ventilation or nephropathy indicating dialysis)	Sepsis or multiple organ failure	–	Persistent edema
Infected lymphocele (Retroperitoneal abscess)	Clinical observation or diagnostic evaluation only; intervention not indicated	Medical management indicated (e.g., antibiotics)	Drainage under local anesthesia or without anesthesia indicated	Intervention under general anesthesia indicated (incision and drainage)	At least one organ failure (e.g., pulmonary disorders requiring mechanical ventilation or nephropathy indicating dialysis)	Sepsis or multiple organ failure	Death	Residual abscess on imaging study, occasional fever or abdominal pain
Infectious cervicitis	Clinical or vaginal observation only; intervention not indicated	Medical management indicated (e.g., antibiotics)	Drainage under local anesthesia or without anesthesia indicated	Intervention under general anesthesia indicated (drainage, hysterectomy)	At least one organ failure (e.g., pulmonary disorders requiring mechanical ventilation or nephropathy indicating dialysis)	Sepsis or multiple organ failure	Death	Persistent infected vaginal discharge
Uterine infection	Clinical observation or diagnostic evaluation only; intervention not indicated	Medical management indicated (e.g., antibiotics)	Dilation and curettage under local anesthesia or without anesthesia indicated	Intervention under general anesthesia indicated (drainage, hysterectomy)	At least one organ failure (e.g., pulmonary disorders requiring mechanical ventilation or nephropathy indicating dialysis)	Sepsis or multiple organ failure	Death	Residual abscess on imaging study, occasional fever or abdominal pain
Ovarian infection	Clinical observation or diagnostic evaluation only; intervention not indicated	Medical management indicated (e.g., antibiotics)	Paracentesis drainage under local anesthesia indicated	Intervention under general anesthesia indicated (drainage, oophorectomy)	At least one organ failure (e.g., pulmonary disorders requiring mechanical ventilation or nephropathy indicating dialysis)	Sepsis or multiple organ failure	Death	Residual abscess on imaging study, occasional fever or abdominal pain
Vulval infection	Clinical observation or diagnostic evaluation only; intervention not indicated	Medical management indicated (e.g., antibiotics)	Paracentesis drainage under local anesthesia indicated	Intervention under general anesthesia indicated (drainage, skin flap, or musculocutaneous flap)	At least one organ failure (e.g., pulmonary disorders requiring mechanical ventilation or nephropathy indicating dialysis)	Sepsis or multiple organ failure	Death	Residual abscess on imaging study, occasional fever or abdominal pain
Wound infection	Clinical observation or diagnostic evaluation only; intervention not indicated, except for wound opening and wound irrigation at the bedside	Medical management indicated (e.g., antibiotics)	Medical intervention under local anesthesia indicated (e.g., drainage)	Intervention under general anesthesia indicated (e.g., drainage, resuturing)	At least one organ failure (e.g., pulmonary disorders requiring mechanical ventilation or nephropathy indicating dialysis)	Sepsis or multiple organ failure	Death	Continued outpatient irrigation
Implant infection	Clinical observation or diagnostic evaluation only; intervention not indicated	Medical management indicated (e.g., antibiotics)	Medical intervention under local anesthesia indicated (e.g., incision and drainage, implant removal)	Intervention under general anesthesia indicated (implant removal)	At least one organ failure (e.g., pulmonary disorders requiring mechanical ventilation or nephropathy indicating dialysis)	Sepsis or multiple organ failure	Death	Discharged with drainage tube placement; persistent infection
Bladder injury	Foley catheter placement indicated	Medical management indicated (e.g., antibiotics)	–	Intervention under general anesthesia indicated	At least one organ failure (e.g., pulmonary disorders requiring mechanical ventilation or nephropathy indicating dialysis)	Sepsis or multiple organ failure	Death	Discharged with Foley catheter placement
Urinary incontinence	Intermittent catheterization or Foley catheter placement indicated	Medical management indicated (e.g., anticholinergics)	Intervention under local or lumbar anesthesia indicated (e.g., clamp, collagen injection)	Intervention under general anesthesia indicated (e.g., artificial urinary sphincter)	Acute renal failure, hemodialysis	Sepsis or multiple organ failure	Death	Persistent condition requiring Intermittent catheterization; Discharged with Foley catheter placement
Residual urine/Urinary retention	Intermittent catheterization or Foley catheter placement indicated	Medical management indicated (e.g., cholinergics)	Intervention under local or lumbar anesthesia indicated (e.g., endoscopic treatment, urethral dilatation)	Intervention under general anesthesia indicated (e.g., fistula closure)	Acute renal failure, hemodialysis	Sepsis or multiple organ failure	Death	Persistent condition requiring intermittent catheterization; Discharged with Foley catheter placement
Dyspareunia	Discomfort associated with vaginal penetration; intervention not indicated	Estrogen administration indicated	Medical intervention under local anesthesia indicated	Intervention under general anesthesia indicated	–	–	–	Persistent pain associated with sexual intercourse, persistent dyspareunia
Erectile dysfunction	Erectile dysfunction; intervention not indicated, except for external vacuum device for managing erectile dysfunction	Medical management indicated (e.g., Phosphodiesterase 5 inhibitors or intracavernosal injection of vasoactive agonists)	Intervention under local or lumbar anesthesia indicated	Intervention under general anesthesia indicated (e.g., penile prosthesis)	–	–	–	Persistent erectile dysfunction
Cervical atresia (uterine atresia)	Clinical or vaginal observation only; intervention not indicated	Associated with dysmenorrhea; medical management indicated (e.g., analgesics)	Bougienage of cervical duct with or without local anesthesia indicated	Intervention under general anesthesia indicated (cervical dilatation)	–	–	–	Persistent stenosis of the cervical os
Vaginal fistula	Clinical or vaginal observation only; intervention not indicated	Medical management indicated (e.g., antibiotics)	–	Intervention under general anesthesia indicated (vaginal fistula closure, colostomy)	At least one organ failure (e.g., pulmonary disorders requiring mechanical ventilation or nephropathy indicating dialysis)	Sepsis or multiple organ failure	Death	Persistent leakage from vagina
Ovarian deficiency syndrome	Clinical observation or diagnostic evaluation only; intervention not indicated	Medical management indicated (e.g., hormone replacement therapy)	–	–	–	–	Death	Hot flash requiring continued hormone replacement therapy, depression requiring continued psychiatric care
Cervical chylous leakage	Observation of chylous drainage fluid or paracentesis fluid only; intervention not indicated (drainage only through existing drainage tube)	Fat-restricted diet, intravenous nutrition indicated	Image-guided drain placement/paracentesis including drain replacement indicated.	Intervention under general anesthesia indicated	–	–	Death	Persistent sensation of pressure in the neck
Serous leakage	Clinical observation only; intervention not indicated (drainage only through existing drainage tube)	Medical management indicated (e.g., antibiotics)	Image-guided drain placement/paracentesis including drain replacement indicated	Intervention under general anesthesia indicated	At least one organ failure (e.g., pulmonary disorders requiring mechanical ventilation or nephropathy indicating dialysis)	Sepsis or multiple organ failure	Death	Exudate leakage from the wound, occasional fever and infection, discharged with drainage tube
Chylous ascites	Observation of chylous drainage fluid or paracentesis fluid only; intervention not indicated (drainage only through existing drainage tube)	Fat-restricted diet, intravenous nutrition indicated	Image-guided drain placement/paracentesis including drain replacement indicated	Intervention under general anesthesia indicated	–	–	Death	Persistent abdominal fullness
Subcutaneous phlebitis (Mondor disease)	Clinical observation or diagnostic evaluation only; intervention not indicated except for NSAIDs	Opioid administration, or treatment by pain control specialist indicated	Medical intervention under local anesthesia indicated	Intervention under general anesthesia indicated	–	–	–	Surgical site subcutaneous phlebitis; cord-like mass
Thrombosis/embolism	Clinical observation or diagnostic evaluation only; intervention not indicated	Medical management indicated (e.g., anticoagulants)	Invasive treatment indicated (e.g., thrombus ablation via catheter, IVC filter)	Intervention under general anesthesia indicated (pulmonary artery thrombectomy)	Single organ failure caused by thrombi (e.g., lung, brain, heart)	Multiple organ failure caused by thrombi (e.g., lung, brain, heart)	Death	Dyspnea following pulmonary infarction, paralysis following cerebral infarction
Restricted shoulder joint range of motion	Clinical observation only; intervention not indicated except for NSAIDs	Opioid administration, or treatment by pain control specialist indicated	Surgical intervention without general anesthesia indicated (e.g., nerve block)	Intervention under general anesthesia indicated	–	–	–	Continued restriction in the range of motion of the shoulder joint
Fat necrosis	Clinical observation or diagnostic evaluation only; intervention not indicated except for wound opening and wound irrigation at the bedside	Medical management indicated (e.g., antibiotics)	Medical intervention under local anesthesia indicated (e.g., incision and drainage)	Intervention under general anesthesia indicated	At least one organ failure (e.g., pulmonary disorders requiring mechanical ventilation or nephropathy indicating dialysis)	Sepsis or multiple organ failure	Death	Wound fat necrosis, occasional cicatrization, fever or infection
Skin necrosis (flap necrosis)	Clinical observation or diagnostic evaluation only; intervention not indicated	Medical management indicated (e.g., antibiotics)	Medical intervention under local anesthesia indicated (e.g., debridement, skin grafting)	Intervention under general anesthesia indicated (skin grafting)	At least one organ failure (e.g., pulmonary disorders requiring mechanical ventilation or nephropathy indicating dialysis)	Sepsis or multiple organ failure	Death	Insufficient epithelialization, persistent infection
Subcutaneous emphysema	Clinical observation or diagnostic evaluation only; intervention not indicated except for subcutaneous puncture and compression with breast band at the bedside	–	Radiological intervention treatment without general anesthesia indicated (e.g., subcutaneous drain insertion)	Intervention under general anesthesia indicated	–	–	–	Discharged with subcutaneous air accumulation
Upper extremity edema	Intervention not indicated except for lymphatic massage and elastic stockings	Medical management indicated (e.g., diuretics)	Intervention under local anesthesia indicated (lymphatic anastomosis)	Intervention under general anesthesia indicated (lymphatic anastomosis)	–	–	–	Continued elastic stocking use
Lower extremity lymphedema (edema of the extremities, lymphedema, localized edema)	Intervention not indicated except for lymphatic massage and elastic stockings	Medical management indicated (e.g., diuretics)	Intervention under local anesthesia indicated (lymphatic anastomosis)	Intervention under general anesthesia indicated (lymphatic anastomosis)	–	–	–	Continued elastic stocking use
Obturator/femoral neuropathy (Gait disturbance)	Intervention not indicated except for walking aid and rehabilitation	Medical management indicated (e.g., vitamins)	–	Intervention under general anesthesia indicated (e.g., nerve suture)	–	–	–	Persistent restriction in lower extremity adduction
Wound pain	Clinical observation only; intervention not indicated except for NSAIDs	Opioid administration, or treatment by pain control specialist indicated	Surgical intervention indicated (e.g., nerve block)	–	–	–	–	Home pain control
Others (No AE term)	Deviation from normal postoperative course. Medication, surgical intervention, endoscopic treatment, or radiological intervention treatment not indicated Treatment with antiemetics, antipyretics, analgesics, or diuretics; electrolyte replenishment; or physical therapy is not included in this category (even if these treatments are indicated, the condition is categorized as Grade I); open wound infection at the bedside is Grade I	Medication indicated except for antiemetics, antipyretics, analgesics, and diuretics Cases requiring blood transfusion or intravenous hyperalimentation are included	Surgical, endoscopic, or radiological intervention treatment indicated (without general anesthesia)	Surgical, endoscopic, radiological intervention treatment indicated (intervention under general anesthesia)	IC/ICU management indicated; life-threatening complications (including complications in the central nervous system) AND single organ failure (including dialysis)	IC/ICU management indicated; life-threatening complications (including complications in the central nervous system) AND multiple organ failure	Death	

*IC* intermediate care, *ICU* intensive care unit, *TPN* total parenteral nutrition, *PPI* proton pump inhibitor, *NG tube* nasogastric tube, *CT* computed tomography

**Table 2:** Characteristics of the three criteria

	CTCAE ver4.0	Clavien-Dindo classification	JCOG PC criteria
AE terms	Specified	Not specified	Specified
Grading definitions	Defined for each AE	Single common definition for all AEs	Defined for each AE (following the general definition of the Clavien-Dindo classification)

## Discussion

Until Clavien PA et al. published their original classification in 1992, there were no established criteria or framework available to standardize surgical complications in surgical trials. In 2003, the US National Cancer Institute-Common Toxicity Criteria (NCI-CTC) version 2.0 [9] were revised and renamed the CTCAE version 3.0 [10]. This system has been used widely to evaluate and define the toxicity of chemotherapy or radiotherapy. While terms and definitions for AEs occurring as a result of intraoperative and postoperative complications were not included in the NCI-CTC version 2.0, some surgical AE terms were incorporated in the CTCAE version 3.0. Nevertheless, the CTCAE version 3.0 failed to include many surgical complications and surgeons were frequently unable to objectively classify complications using its grading definitions.

In 2009, the CTCAE version 4.0 [11] was released, with considerably more surgical AE terms, but several common surgical complications were still not included. For example, intra-abdominal abscess, pyothorax, delayed gastric emptying, and lung torsion were not listed as AE terms. Moreover, grading definitions were not clinically optimized for some surgical AEs. For example, the grading definition of pancreatic fistula in this version of the CTCAE is suitable for pancreatitis, but not for pancreatic fistula after pancreatectomy. Such inappropriate definitions have made surgeons reluctant to use the CTCAE version 4.0 in surgical trials.

In 2004, the Clavien-Dindo classification was modified to allow for the grading of life-threatening complications and long-term disability caused by a complication. This revised version defines five grades of severity (Grade I, II, IIIa, IIIb, IVa, IVb, and V) and the suffix “d” (for “disability”) is used to denote any postoperative impairment [7]. This refined Clavien-Dindo classification has been used increasingly in clinical practice and also in clinical trials involving surgical procedures, because it is simple, reproducible, and flexible [12]. Rather than providing specific grading criteria for each AE, the Clavien-Dindo classification provides broad-based but general criteria that can be used uniformly for all kinds of surgical AEs. However, several issues have emerged since this classification became more widely used. One controversial issue is that AE terms are not well defined and different AE terms designate the same AEs in different clinical trials. For example, when intestinal obstruction occurs, some investigators could report this AE as “ileus”, but others refer to it as “small bowel obstruction” or “colon obstruction”. Under such circumstances, the incidence of this AE cannot be counted accurately. A second issue is that only general grading criteria are defined and therefore, grading can be difficult in some cases and subject to bias by the grader. For example, primary non-operative treatment for intestinal obstruction is gastroenteric tube decompression. Nasogastric tube or nasoenteric tube is utilized depending on the severity, but the original Clavien-Dindo classification does not define what grading should be applied for any type of gastroenteric tube placement for decompression.

The JCOG PC criteria were established to address these issues. The advantages of the JCOG PC criteria are as follows: First, commonly experienced surgical AEs are specified and listed. To compare precisely the frequency of surgical complications between studies, use of the common AE terms specified in the JCOG PC criteria is recommended. Second, grading definitions are straightforward and optimized for surgical complications. With these advantages, the JCOG recommends that the JCOG PC criteria be used to supplement the Clavien-Dindo classification, while maintaining the overall Clavien-Dindo classification. In JCOG, some disease-oriented subgroups are conducting clinical trials including surgery and using both the CTCAE and JCOG PC criteria to evaluate postoperative complications. After these trials are completed, we will evaluate the concordance between the grading by the CTCAE and that by the JCOG PC criteria. We also plan to explore the advantages and disadvantages of the JCOG PC criteria.

The JCOG PC criteria have some limitations. First, these AE terms were chosen somewhat arbitrarily, but by experienced surgeons, and specific grading was decided based on the opinions and experience of our committee members. A second limitation of the JCOG PC criteria is that they do not include intraoperative complications. Our intent was to further define and clarify the criteria of the Clavien-Dindo classification and we considered that incorporating intraoperative complications would deviate too much from the original concept. Another common classification may be required to define and grade intraoperative complications. A third limitation is that all descriptions in the Clavien-Dindo classification pertain to early postoperative complications. Here, ‘early postoperative’ generally indicates the time from surgery to the first hospital discharge, but in theory, the Clavien-Dindo classification can be applied broadly to late postoperative complications after hospital discharge. Within this context, the JCOG PC criteria are mainly intended to be used for early postoperative complications, but they can also be used after hospital discharge, although would require more definitions and AEs.

In conclusion, the goals of the JCOG PC criteria are to standardize the AE terms used for early postoperative complications by providing more detailed grading guidelines based on the Clavien-Dindo classification. We suggest that researchers use the JCOG PC criteria in every surgical trial to allow for precise comparison of the frequency of surgical complications among trials.
